# Impact of low molecular weight heparin administration on the clinical course of the COVID-19 disease

**DOI:** 10.3906/sag-2006-184

**Published:** 2021-02-26

**Authors:** Burcu YORMAZ, Dilek ERGÜN, Baykal TÜLEK, Recai ERGÜN, Uğur ARSLAN, Fikret KANAT

**Affiliations:** 1 Department of Pulmonology, Faculty of Medicine, Selçuk University, Konya Turkey; 2 Department of Microbiology, Faculty of Medicine, Selçuk University, Konya Turkey

**Keywords:** Coronavirus disease-19, heparin, antiinflammatory, lymphocyte

## Abstract

**Background:**

Lymphopenia is the most important criterion of mortality and discharging feature for patients infected with coronavirus disease 2019 (COVID-19). This study aimed to investigate the clinical impact of a low molecular weight heparin (LMWH) treatment on the clinical course of COVID-19.

**Materials and methods:**

Patients’ clinical symptoms, radiologic outcomes, hematologic, biochemical, D-dimer, and C-reactive protein (CRP) results were obtained from their medical records. Participants were separated into 2 groups: one was treated with LMWH and the other was not. Improvement in the patients was compared before and after treatment.

**Results:**

Ninety-six patients who were diagnosed with COVID-19 between April and May 2020 were retrospectively analyzed. The multivariable analysis showed that the count of lymphocytes, D-dimer, and CRP levels were significantly improved in the LMWH group, as compared to the control group (OR, (95% CI) 0.628 (0.248–0.965), P < 0.001); OR, (95% CI) 0.356 (0.089–0.674), P < 0.001, respectively). The area under the receiver operating characteristic (ROC) curve analysis was AUC: 0.679 ± 0.055, 0.615 ± 0.058, and 0.633 ± 0.057, respectively; the β-value was found to be –1.032, –0.026, and –0.465, respectively.

**Conclusion:**

The LMWH treatment group demonstrated better laboratory findings, including recovery in the lymphocyte count, CRP, and D-dimer results.

## 1. Introduction

The COVID-19 disease is an illness caused by a respiratory and systemic zoonotic coronavirus. It was first recognized in Wuhan, China, and it has continued to spread rapidly since then. It has become a global calamity, and the World Health Organization (WHO) declared the disease a pandemic on March 11, 2020. Over 2 million people worldwide have been infected so far, and mortality continues to rise. However, no effective medical cure is available, and patients are treated according to their symptoms and findings [1,2].

Helpful strategies for improvement of the illness may be devised if the pathophysiology is understood. Lymphopenia and cytokine storms are the typical pathological changes detected in patients with coronavirus infections; they relate to COVID-19 severity. The cytokine storm is a key mechanism underlying disease exacerbation and mortality in COVID-19 patients [3–5]. Some studies have indicated that low molecular weight heparin (LMWH) has some properties other than its anticoagulant effects, such as its antiinflammatory action, which ensures improvement in lymphopenia [6]. However, although the antiinflammatory effects of LMWH in COVID-19 remain unclear, it is thought that the antiinflammatory efficacy contributes to the regression of COVID-19 [7]. The aim of this study is to assess the clinical impact of LMWH treatment on the clinical course of COVID-19.

## 2. Material and methods

In this study, 96 participants who were admitted to the Selcuk University Hospital, Department of Pulmonology between March and April 2020 were analyzed retrospectively. The patients’ clinical outcomes were investigated by studying their electronic medical records. COVID-19 was diagnosed according to WHO guidelines. This study was approved by the Ministry of Health Committee (approval number: 2020/37) and conducted in accordance with the principles of the Declaration of Helsinki [2].

Blood samples were gathered during the length of the stay in the hospital: D-dimer, prothrombin time (PT), international normalized ratio (INR), and fibrinogen (FIB) measurements were investigated by utilizing a coagulation analyzer device.

Participants were separated into 2 groups according to D-dimer (D-dimer > 750 ng/mL) and PT (<12 s) outcomes due to mortality, raised accordingly with D-dimer, PT levels, and given appropriate thrombopropylaxis with LMWH (a thromboprophylactic dose of 4000 UI/day for 7 days). No anticoagulant drugs other than heparin were utilized for 7 days or longer in the research patients. All the participants received appropriate treatment after admission to the hospital [8–10].

### 2.1. Inclusion criteria

The inclusion criteria included: 1) satisfying the conditions of the diagnostic standards of COVID-19 pneumonia outlined by the Health Commission of Turkey, 2) identified as tightness of breath, respiration rate (RR) ≥ 30/min, detected typical lesions in CT images of viral pneumonia; 3) age ≥18 years; 4) no history of any pulmonary disease; and 5) no immunosuppressive or corticosteroid agent administered during the therapy period.

### 2.2. Exclusion criteria

The exclusion criteria included: 1) participants with severe chronic diseases; 2) patients who had liver, kidney, or cardiac disease; 3) patients who had taken LMWH therapy in the last 3 months; 4) patients with a history of mental disorders; 5) women who were pregnant or breastfeeding; 6) patients who had followed-up in the intensive care unit (ICU); and 7) patients with any sensitivity to LMWH.

### 2.3. Chest CT severity score assessment

The computer tomography severity score (CT-SS) was utilized to evaluate patients with COVID-19. The CT-SS is an adapted version of the scoring system, describing ground glass, interstitial opacity, and air trapping for severe acute respiratory syndrome (SARS). This scoring system assesses pulmonary lesions as a guide for detecting the disease [11]. The lungs were separated into 20 zones, according to human anatomy. The detected pulmonary opacities in all zones were assessed by thin section thorax CT (TST-CT), using system linked scores of 0, 1, and 2, according to opacification, including 0%, < 50%, or ≥ 50% of every zone. The total scores, which ranged between 0 and 40 points, were obtained from each zone collected. Two experienced thorax radiologists, who were blinded to the identity of the participants, evaluated all the CT screenings. Chest CT scans were performed with a 256-detector CT scanner (Siemens, Germany). All the participants were lying in the supine position, and the scan was performed during the breath hold situation [11].

### 2.4. Data collection

The demographic characteristics (age, sex, and BMI), signs and symptoms, clinical outcomes, initial knowledge, complete blood count (CBC), D-dimer, FIB, coagulation profile, inflammatory markers, biochemical markers (such as liver function, CRP, and electrolytes), and the TST-CT of patients infected with COVID-19 were assessed retrospectively (Tables 1–3). Two researchers assessed the collected data forms independently.

**Table 1:** General characteristics of the patients infected with COVID-19.


	LMWH Group(n = 48)	Control Group (n = 48)	P-value
Characteristics			
Age (years)	53.3 ± 15.6	55.4 ± 11.6	0.469
Sex			0.667
Female	15 (31.3)	18 (37.5)	
Male	33 (68.8)	30 (62.5)	
Comorbidity	29 (60.4)	28 (58.3)	0.718
Hypertension	17 (35.4)	18 (37.5)	0.617
Diabetes mellitus	14 (29.2)	10 (20.8)	0.123
Coronary artery disease	7 (12.2)	5 (8.7)	0.831
Gastrointestinal disease	1 (2.1)	2 (4.2)	0.512
Other disease	6 (12.5)	3 (6.3)	0.486

Values were presented as mean ± standard deviation (min–max), median (min–max) or numbers (n) and percentages (%). P-values were calculated by Welch’s t-test, Mann–Whitney U test, χ2 test with Yates continuity correction or Fisher’s exact test, as appropriate.

**Table 2:** Signs, symptoms, and clinical outcomes.

	LMWH Group(n = 48)	Control(n = 48)	P-value
Signs			
Fever (temperature ≥37°C)	38 (79.1%)	36 (75.0%)	0.808
Dry cough	33 (68.8%)	30 (62.5%)	0.667
Shortness of breath	24 (50.0%)	28 (58.3%)	0.413
Sputum	13 (27.1%)	14 (29.2%)	0.825
Myalgia	10 (20.8%)	16 (33.3%)	0.251
Throat ache	8 (16.7%)	6 (12.5%)	0.772
Nausea or vomiting	1 (2.1%)	6 (12.5%)	0.135
Diarrhea	3 (6.9%)	4 (8.3%)	0.683
Headache	4 (8.3%)	2 (4.2%)	0.277
Time from initial symptoms to admission to hospital (days)	2.2(1.1–3.4)	2.4(1.6–3.2)	0.816
Time from hospitalization to viral shedding of disease (days)	5.2 (3.4–6.3)	7.6 (6.5–9.7)	<0.001
Length of stay in hospital	7.2 (6.4–8.3)	9.6 (8.5–10.7)	<0.001
Antibiotic therapy	48	48	0.997
Azitromycin	38 (79.1%)	39 (81.2%)	
Moxifloxacin	42 (87.5%)	41 (85.4%)	
Antiviral therapy			0.994
Favipiravir	32 (66.6%)	33 (68.7%)	
Oseltamivir	43 (89.5%)	42 (87.5%)	
Response to treatment			
Improved	48 (100%)	30 (62.5%)	0.212
Steady	0	18 (37.5%)	
Disruptive	0	0	
CT-SS			
Score of left lung	5.0 (4.0–6.0)	6.0 (5.0–8.0)	<0.001
Score of right lung	5.0 (3.75–6.0)	7.5 (6.0–95)	<0.001
Total score	11.0 (7.0–12.5)	13.5 (12.5–16.0)	<0.001
CT-SS: CT severity score			

**Table 3:** Laboratory findings and scores during the treatment period.

	Initial values			7th day of the treatment			
	LMWH(n = 48)	Control(n = 48)	pa	LMWH(n = 48)	Control(n = 48)	Pb	Pc
WBC (k/uL)	9.26 ± 0.76	9.51 ± 0.80	0.497	6.40 ± 0.41	6.91 ± 0.37	0.365	0.435
Neutrophil (k/uL)	5.43 ± 0.54	4.88 ± 0.43	0.402	4.95 ± 0.45	4.84 ± 0.32	0.828	0.408
Monocyte (k/uL)	0.55 ± 0.05	0.52 ± 0.04	0.609	0.58 ± 0.03	0.65 ± 0.05	0.260	0.523
Monocyte percent (%)	7.39 ± 0.49	7.93 ± 0.64	0.491	7.87 ± 0.53	8.99 ± 0.67	0.175	0.318
Lymphocyte (k/uL)1,39	0.77 ± 0.03	0.82 ± 0.02	0.094	1.39 ± 0.40	1.02 ± 0.03	<0.001	<0.001
Lymphocytes percent (%)	12.41 ± 1.06	13.66 ± 0.84	0.205	22.18 ± 2.02	19.86 ± 1.46	0.337	<0.001
HB (g/dL)	12.80 ± 0.35	13.29 ± 0.27	0.242	12.31 ± 0.31	12.96 ± 0.25	0.089	0.001
PLT (k/uL)	181.72 ± 11.83	189.05 ± 9.58	0.618	220.60 ± 11.17	229.15 ± 13.28	0.610	<0.001
ALT (U/L)	44.12 ± 5.26	43.63 ± 6.23	0.879	35.16 ± 3.64	36.17 ± 4.28	0.826	0.853
AST( U/L)	42.27 ± 3.45	43.72 ± 4.48	0.911	37.83 ± 4.95	35.82 ± 3.59	0.868	0.889
TB (mmol/L)	1.04 ± 0.13	1.06 ± 0.24	0.752	0.98 ± 0.26	1.00 ± 0.14	0.842	0.892
Na	138.25 ± 3.45	141.40 ± 2.48	0.764	142.26 ± 3.25	144.52 ± 4.28	0.815	0.795
K	4.32 ± 1.13	4.46 ± 1.48	0.827	4.53 ± 1.26	4.61 ± 2.01	0.874	0.855
Cre	1.10 ± 0.25	1.16 ± 0.44	0.745	0.98 ± 0.55	1.01 ± 0.62	0.825	0.794
CK-MB (ng/mL)	1.22 ± 0.15	1.28 ± 0.11	0.738	1.25 ± 0.15	1.33 ± 0.12	0.663	0.827
Troponin-I (ng/L)	9.46 ± 2.56	5.71 ± 0.79	0.152	9.06 ± 2.33	4.84 ± 0.99	0.089	0.750
PT (sn)	11.16 ± 0.15	13.36 ± 0.31	0.256	15.05 ± 0.13	11.78 ± 0.38	<0.001	<0.001
INR (INR)	1.03 ± 0.02	1.23 ± 0.03	0.540	1.43 ± 0.02	1.11 ± 0.04	0.069	0.229
D-DIMER (ng/mL) < 414	815.02 ± 112.24	650 ± 59.68	0.182	414.10 ± 45.73	635.63 ± 39.96	<0.001	<0.001
CRP (mg/L)	43.66 ± 9.93	41.76 ± 7.20	0.134	14.32 ± 4.46	36.62 ± 3.09	<0.001	<0.001
FIB	588.24 ± 10.25	595.35 ± 12.36	0.627	326.35 ± 12.25	550.37 ± 15.38	<0.001	<0.001
CT-SS	15.0 (12.0–16.0)	15.3 (13.5–16.5)	0.983	11.0 (7.0–12.5)	13.5 (12.5–16.0)	<0.001	<0.001


Values were presented as trimmed mean ± SEM (trimmed mean was calculated with 10% trim proportion); (SEM: standard error of mean). P-values were calculated with Yuen’s test (robust independent samples t-test) and robust paired samples t-test. P^a^ shows the comparison between LMWH and control groups before the treatment. P^b^ shows the comparison between LMWH and control groups on the 7th day of the treatment. P^c^ shows the comparison between before and on the 7th day of treatment in the LMWH group. Abbreviations: WBC: white blood cell; HB: hemoglobin; PLT: platelat; CK-MB: creatinine kinase–myocardial band isoenzyme; PT: prothrombin time; INR: international normalized ratio; CRP: C-reactive protein; FIB: fibrinogen; ALT: alanineaminotransferase; AST: aspartateaminotransferase; TB: total bilirubine, K: potassium; Na: sodium, Cre: creatinine.

### 2.5. Statistical analysis

**Figure 1:**
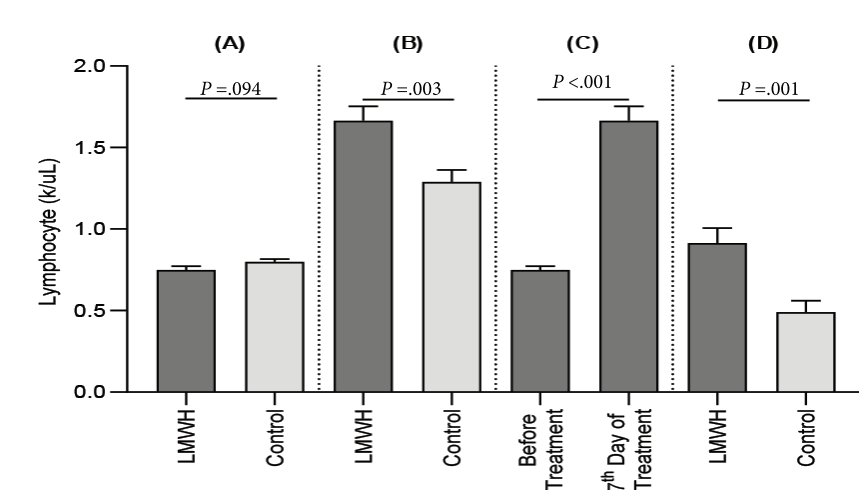
Effect of LMWH on lymphocyte (k/uL) in the patients with COVID-19. Data were expressed as mean ± SEM. (A): Comparison between LMWH and control groups before the treatment; (B): Comparison between LMWH and control groups on the 7th day of the treatment. (C): Comparison between before and on the 7th day of the treatment in the LMWH group. (D): Comparison between the changes, which were calculated by taking the difference between the 7th day results and the results collected before the treatment in both groups.

**Figure 2:**
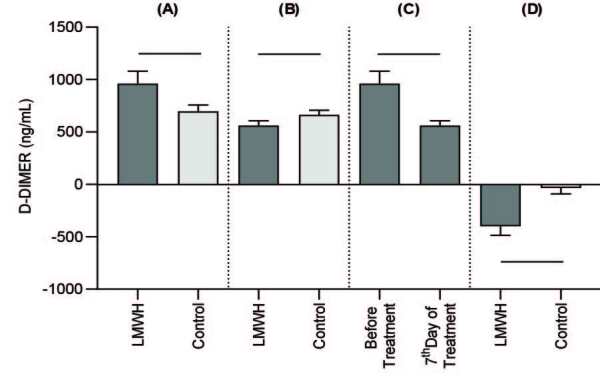
Effect of LMWH on D-Dimer (ng/mL) in the patients with COVID-19. Data were expressed as mean ± SEM. (A): Comparison between LMWH and control groups before the treatment. (B): Comparison between the LMWH and control groups on the 7th day of treatment. (C): Comparison between before and on the 7th day of the treatment in the LMWH group. (D): Comparison between the changes, which were calculated by taking the difference between the 7th day results and results collected before treatment in both groups.

The R 3.6.0 (www.r-project.com) was used to perform all the statistical analyses. The Anderson–Darling test and Q-Q plots were used to check the normality of the variables. The homogeneity of the variances by group was examined using the Levene’s test. Data was described as mean ± standard deviation (range), median (interquartile range), and numbers (%) for the general characteristics of the patients with COVID-19. Welch’s t-test, the Mann–Whitney U test, the *χ* 2 test, with the Yates continuity correction, and Fisher’s exact test were used, as appropriate, to compare the general characteristics between the patient groups. Considering the possible extreme outliers under pandemic conditions, the values for the laboratory findings were presented as trimmed mean (±SEM: standard error of mean), which was calculated with a 10% trim proportion and a robust estimator against the outliers. Yuen’s test (robust independent-samples t-test) and the robust paired-samples t-test were used to compare these findings. The comparisons were applied considering 4 situations: the LMWH and the control groups before treatment, the LMWH and the control groups on the 7th day of treatment, and the LMWH group before treatment and on the 7th day of treatment (Figures 1 and 2). Finally, calculated changes were compared by taking the difference of the 7th day and before the treatment in both groups; P < 0.001 was considered statistically significant. Univariate logistic regression analysis was utilized to view risk factors. A multivariate logistic regression analysis was performed to evaluate the efficacy of the variant risk factors on the participant’s discharge and scoring system; the odds ratio (OR) and 95% confidence interval (CI) were calculated (Tables 4,5). The forecasting value of the lymphocyte count, D-dimer, and CRP, as assessed by the ROC curve and the cut-off value, which may predict the discharge, were identified afterward. 

**Table 4:** Univariate logistic regression analysis of risk factors with COVID-19 improvement.

	UnivariableOR (95% CI)	P value
Age	1.041 (1.018–1.057)	0.315
Sex	0.732 (0.457–1.104)	0.217
BMI (kg/m2)	0.538 (0.319–0.936)	0.472
CT-SS	12.30 (10.50–14.25)	<0.001
Laboratory findings		
WBC (k/uL)	2.485 (1.821–3.178)	<0.001
Neutrophils (k/uL)	1.514 (1.137–1.749)	<0.001
Lymphocyte (k/uL)	0.203 (0.015–0.322)	<0.001
Antiinflammatory markers		
CRP (mg/L)	0.362 (01.131–0.926)	<0.001
D-DIMER (ng/mL)	0.657 (0.463–1.223)	<0.001
Fibrinogen	1.738 (1.176–2.549)	<0.001
CT-SS: CT severity score, CRP: C reactive protein.		

**Table 5:** Multivariate logistic regression analysis of risk factors with COVID-19 improvement.

	β	OR (95% CI)	P
Lymphocyte (k/uL)	–1.032	0.356 (0.089–0.674)	<0.001
CRP (mg/L)	–0.465	0.628 (0.248–0.965)	<0.001
D-DIMER (ng/mL)	–0.026	0.974 (0.476–1.594)	<0.001

### 2.6. ROC curve analysis

**Figure 3:**
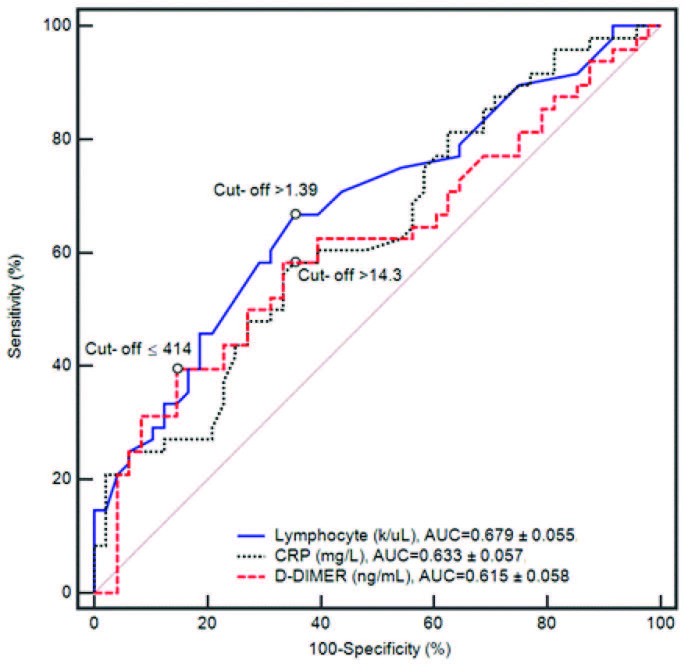
ROC curve for risk factors in patients infected with COVID-19. ROC = receiver operating characteristic.

The recovery performances of the laboratory parameters were evaluated by ROC analysis on the 7th day, as shown in Figure 3. The cut-off point for these parameters was determined according to the Youden index value. Risk factors included the lymphocyte counts, the level of CRP, and the D-dimer. The contributions of the risk factors were determined based on the β value presented in Table 5. The area under the ROC curve for dividing the participants’ LMWH, as compared to the control group, was applied for the threshold sensitivity, specificity, and accuracy. It was OR; 0.356 (standard error, 0.001; 95% CI, 0.089–0.674, β; –1.032) for lymphocyte, OR; 0.974 (standard error, 0.001; 95% CI, 0.476–1.594, β; –0.026) for D-dimer, and OR; 0.628 (standard error, 0.001; 95% CI, 0.248–0.965, β; –0.465) for CRP. The optimal threshold for identifying patients was 1.39, with 66.7% sensitivity and 64.6% specificity; 414, with 39.6% sensitivity and 85.4% specificity; and 14.3, with 58.3% sensitivity and 64.6% specificity, respectively.

## 3. Results

### 3.1. General characteristics of the patients with COVID-19

The LMWH group consisted of 15 males and 33 females aged between 40 and 68 years (average age: 53.3 years), as shown in Table 1. The control group consisted of 18 males and 30 females aged between 44 and 66 years (average age: 55.4 years). There was no substantial difference detected between the groups.

There were no significant differences between the groups in comorbidities such as diabetes mellitus, hypertension, coronary artery disease, gastrointestinal disease, cardiovascular disease, or other diseases. There were also no significant differences in COVID-19 pneumonia onset symptoms, with the inclusion of fever (temperature ≥37.4 °C), dry cough, shortness of breath, sputum, myalgia, throat ache, nausea or vomiting, diarrhea, and headache. Similarly, there was no significant difference in the conventional therapy between the groups. These outcomes show that the general characteristics of the patient groups were both congruous and comparable.

### 3.2. Effect of LMWH on the days to conversion to negative and the length of the hospital stay of patients with COVID-19

As shown in Table 2, the number of days it takes to convert the virus to a negative outcome (time from the beginning of the stay in the hospital until virus shedding) was 5.2 days (IQR 3.4–6.3) in the LMWH group and 7.6 days (IQR 6.5–9.7) in the control group (P < 0.001). A significant difference was detected between the groups. Also, the length of the stay in the hospital was 7.2 days (IQR 6.4–8.3) in the heparin group and 9.6 days (IQR 8.5–10.7) in the control group (P < 0.001). A significant difference was detected between the groups. All of the participants demonstrated improvement after the treatment.

### 3.3. Effect of LMWH on the blood routine in patients with COVID-19

A significant difference was detected in the lymphocyte count between the groups before and after treatment, as shown in Table 2. The after-treatment lymphocyte count of the LMWH treatment group participants was elevated, and the detected difference was significant. In addition, the changes in the lymphocyte counts in the LMWH group before and after therapy were significantly different according to the control group. 

After treatment, the platelet results and levels were significantly elevated in the LMWH group, compared to the control group. However, there was no significant difference detected between the groups in the monocyte, neutrophil percent, white blood cell (WBC), or hemoglobin levels.

### 3.4. Effect of LMWH on the coagulation function in patients with COVID-19

The levels of the D-dimer and fibrin products prior to the application of LMWH in both groups were nonsignificant; however, these outcomes were significantly decreased after the LMWH treatment in the LMWH group, compared to the control group, as shown in Table 3. The patients’ levels of D-dimer and FIB were significantly decreased in the LMWH group prior to and after treatment. The outcomes show that the application of LMWH improves the hypercoagulable state in COVID-19 patients. However, there was no significant difference detected in PT and INR levels among the groups.

### 3.5. Effect of LMWH on the CRP in patients with COVID-19

There were significant differences detected in the CRP levels between the groups both prior to and after LMWH treatment, as shown in Table 3. Although the CRP levels were initially similar between the groups, these outcomes were significantly decreased in the LMWH group.

### 3.6. Effect of LMWH on the cardiac markers in patients with COVID-19

As shown in Table 3, there were no significant differences detected in the creatine kinase isoenzymeB (CK-MB) levels between the groups prior to and after LMWH treatment, and the results of both groups were decreased, as compared to prior to treatment. Similarly, there were no significant differences detected in the troponin-I levels between the 2 groups.

### 3.7. Effect of LMWH on the CT-SS in patients with COVID-19

As shown in Table 3, there were significant differences detected in the CT-SS between the groups prior to and after LMWH treatment. Although the CT-SS was initially similar between the groups, these outcomes decreased significantly after LMWH treatment in the LMWH group, as compared to the control group.

## 4. Discussion

Cytokine storms are related to corruption in infectious illnesses, such as SARS and Middle East respiratory syndrome coronavirus (MERS); they are also an important cause of exacerbation in patients. Studies have revealed that heparin has some specifications other than anticoagulant properties. It performs antiinflammatory actions by decreasing the extrication and biological efficacy of IL-6. However, the antiinflammatory effects of heparin in the COVID-19 disease are not fully known. The main finding of this study is to evaluate the impact of LMWH administration on the clinical course of the COVID-19 disease [12].

Although some studies have investigated the nonanticoagulant efficacy of LMWH, the real impact mechanism of LMWH remains unknown. This study investigated the antiinflammatory effect of the LMWH molecule and its contribution to improvement in the COVID-19 disease. LMWH is a heterogeneous molecule, and it has some features other than its anticoagulant effects. One of the effects of LMWH application is that it leads to a decline in inflammation. LMWH efficacy starts with inhibition of leukocyte transmigration stages into tissue. Heparin inhibits inflammation by its anticoagulant efficacy; these properties are closely intertwined. Some studies have investigated its antiinflammatory effect. In an experimental model, Downing et al. found that LMWH reduced inflammation and performed this efficacy without any anticoagulant effects. Ahmed et al. also showed that inhaled heparin led to an improvement in pulmonary functions and airway hypersensitivity in asthmatic patients [13,14].

Although the initial results were similar in the assay of distinctions of the lymphocyte counts, after the therapy period, the lymphocyte counts were more elevated in the LMWH group than the control group, which is consistent with Huang et al. This shows that LMWH can elevate the lymphocyte count and contribute to an improvement of the disease. There are some causes for this. First, LMWH consists mainly of oligosaccharides, and it can be explained that short oligosaccharide chains, which are common in LMWH, may lead to the compression of cytokine storms; in addition, oligosaccharide chains, which are reproduced from nitrous acid depolymerization of LMWH, are able to bind to antithrombin-III (AT-III), and this indicates that the therapeutic effect for the hypersensitivity of LMWH is independent of the anticoagulant impact [15–21].

One large trial on sepsis demonstrated that LMWH decreased mortality rates. This suggests that LMWH regressed the inflammation by its nonanticoagulant effects. Camprubi-Rimblas et al. revealed that LMWH may treat acute respiratory distress syndrome (ARDS) by improving lung inflammation. In addition, Paulsson et al. showed that heparin might heal a lung infection by struggling with heparan sulfate, which can cause a cytokine storm in COVID-19 by preventing the pathogens that bind host cells. However, the RASTEN study revealed that there was no significance detected in the survival time in lung cancer patients; although this was a disappointing outcome for LMWH, the dose of the molecule in this study was prophylactic, and the stage of the participants was higher; therefore, these results are incongruous [22–24].

Researchers and medical personnel have found that age and comorbidity are possible risk factors for patients who are infected with COVID-19. Moreover, some studies have demonstrated that thorax CT scans and laboratory markers, such as complete blood count, liver enzyme markers, coagulation parameters, inflammatory markers, and the quantity of immune cells of COVID-19 patients, are connected with the severity of the illness [25–27].

In some studies on COVID-19, the D-dimer levels were increased substantially according to disease severity. Tang et al. found that elevation of the D-dimer outcomes was correlated with mortality. Furthermore, Zhang et al. detected that elevated results of D-dimer affects hospital mortality, so this is a helpful parameter for following up the improvement of COVID-19 patients. Therefore, D-dimer was used as an evaluation index marker for the progression of the illness in this research. The mean outcomes of D-dimer were higher in the LMWH group than in the control group. This outcome is in line with the achievement of the LMWH performed group. The determination of the differences demonstrated that LMWH had better efficacy for lowering D-dimer levels [11,28].

Novel studies have suggested that analyzing the CRP and lymphocyte counts revealed the efficacy of treatment of COVID-19. When we touched on the differences between the groups, significant differences were detected in the decreasing of the CRP results and the elevation in the lymphocyte counts of the LMWH group, as compared to the control group [29,30].

In the study analysis of CRP outcomes, Walters et al. indicated that LMWH reduces CRP outcomes, indicating its antiinflammatory effect. Furthermore, the ARMADA study revealed that inflammatory markers such as CRP were decreased in the heparin group. According to the control group, the CRP outcomes in the present study were lower in the LMWH group after the therapy period, which is in line with Walters et al. and the ARMADA study. This showed that LMWH can improve CRP levels and contribute to improvement of the disease [31,32].

Novel studies have also investigated some pathobiological perspectives, such as acquired thrombophilia in COVID-19, which were not evaluated previously and may enlighten future research. Patients with COVID-19 frequently have a higher risk for thrombotic situations. Therewithal, fibrin-based occlusions in microvessels have been found in the lung histopathology specimens examined in deceased COVID-19 patients [33–35]. 

Some synergistic mechanisms such as hypoxic vasoocclusion, activation of cells by viral transduction, and cytokine storms, which have been detected in COVID-19, may lead to micro- and/or macrothrombosis [36,37]. Furthermore, numerous studies have demonstrated that activated neutrophils conduce the spreading of thrombus, which affects vascular beds [38,39].

In accordance with these pathophysiological outcomes, some conventional gargling drugs suggested in the initial treatment of COVID-19 for leading oropharyngeal viral shedding, such as *Ankaferd hemostat* , contain *Glycyrrhiza glabra* , which contributes to antiinflammatory efficacy by way of a decline in the high mobility group box 1 protein [HMGB1] secretion [40].

Through the logistic univariate regression model, this study detected that WBC outcomes, the lymphocytes count, neutrophils, D-dimer, FIB, CRP, and PLT were independent risk factors for participants. Moreover, some studies have shown that most of the patients infected with COVID-19 displayed lymphocytopenia, elevated D-dimer levels, CRP, and, in some cases, elevated liver enzymes such as AST and ALT. Hematologic and biochemical outcomes were seriously elevated in patients who were followed up at an ICU, recommending that the severity of the disease may have a relationship with cytokine storms and hyper inflammation. In addition, the logistic multivariate analysis model demonstrated that D-dimer, CRP levels, and lymphocyte counts were the main risk factors for disease severity, which has a relationship with inflammation and hypercoagulation [41,42] **.**

Although some scoring modules have more variables correlated with disease prognosis, no scoring system has been accepted as a rule. Gong et al. formulated a seven-parameter system that included complete blood count and biochemical markers for identification of severity [43]. Chen et al. also performed a system for predicting the prognosis of the disease, including comorbidities, antiinflammatory markers, and demographics [44]. The present study utilized the scoring system designed by Dong et al. for evaluating disease severity and assessing the treatment time by a simple formula, in comparison to other systems that are confusing and may lead to misunderstandings [45]. Although the present system only contains 3 variables, it has better efficacy for distinguishing participants whose progress may turn to severity and respond to treatment conveniently. CRP was used as a scoring parameter, instead of the erythrocyte sedimentation rate (ESR), for evaluating antiinflammatory efficacy more clearly [46]. In comparison to these studies, the present scoring system is a simple and rapid detecting module for patients whose prognosis may become worse.

In addition to the scoring system, this research assessed the TST-CT outcomes for evaluating the improvement of patients and used it to assist in observing the improvement in the lungs. TST-CT is a sensitive apparatus and is better than chest x-rays for investigating the pulmonary parenchyma. Therefore, this method has become a pioneer diagnostic, and it is a helpful method for early detection of COVID-19. The characteristic radiological outcome of COVID-19 is ground-glass opacities and/or consolidation asymmetrically located at peripheral lodges. The radiological manifestations are similar to those of SARS and MERS. The SARS and MERS diseases both frequently show single lesions unilaterally and asymmetrically in the lungs. The TST-CT scores and the screened lesions were improved at the end of the follow-up period, according to the initial time outcomes [47,48].

This study had some limitations. First, it was a retrospective study, and the patient population was small; however, the sample size was considered adequate to draw relevant conclusions. The present study showed that research groups have an inclusive population treated in our center. Second, none of the patients who were treated in the ICU joined the study; all the participants were discharged and had no complications or mortality detected. Finally, the influence of other novel therapies on these patients was not evaluated. Because the research period lasted a little over 1 week, it is possible that some nonpharmacological changes were introduced in the management of patients as medical institutes learned more about this disease over time.

## 5. Conclusion

In conclusion, this research suggests that LMWH therapy, added to conventional treatment, can contribute to clinical and laboratory improvement in COVID-19. Those improvements might be the result of the antiinflammatory effects of LMWH. 

## Informed consent

This research was approved by the Ministry of Health Committee (approval number: 2020/37) and conducted in accordance with the principles of the Declaration of Helsinki (2).

## Ethical Approval

The University Hospital of Medicine faculty approved the study (COVID-19–2020, No. 2020/037). This study was performed in accordance with the Helsinki Declaration of 1964 and its subsequent amendments.
